# Unforeseen consequences: A case report of misdiagnosis in pediatric ingestion of a fish bone

**DOI:** 10.1016/j.radcr.2024.07.071

**Published:** 2024-08-10

**Authors:** Abubakr Bajaber, Safa Shariff, Muhammad Azhar, Mariam Ayashi, Mohammed Moawed, Omar Bajaber

**Affiliations:** a College of Medicine, Alfaisal University, Riyadh 11533, Saudi Arabia; b College of Medicine, Al-Rayan Colleges, Madinah 42541, Saudi Arabia; c Pediatric Surgery Department, King Saud Medical City, Riyadh 12746, Saudi Arabia; d Medical Imaging Department, Pediatric Radiology Section, King Saud Medical City, Riyadh 12746, Saudi Arabia

**Keywords:** Foreign body, Fish bone, Perforation, Abscess, Pediatric, Surgery, Case report

## Abstract

Children are frequently reported as cases of foreign body ingestion, with fishbone ingestion being particularly prevalent in communities where fish consumption is common. Although many instances of foreign body ingestion resolve spontaneously, the ingestion of sharp objects like fishbones poses a greater risk of morbidity and mortality due to their propensity for causing complications. Furthermore, incidents of foreign body ingestion often present with nonspecific symptoms or may go unnoticed, potentially leading to misdiagnosis and complicating the clinical course. We present a case of a 2-year-old boy initially misdiagnosed with constipation and treated with laxatives due to intermittent progressive abdominal pain. Subsequently, he presented to the emergency department where radiological and laboratory investigations revealed signs of inflammation and localized abdominal fluid collection containing a linear hyperdense object, indicating complicated foreign body ingestion with perforation. Urgent laparotomy revealed an omental abscess, which was excised, and the perforation site was repaired with sutures. This case underscores the risk of misdiagnosis and the importance of timely recognition and management. It also emphasizes the critical role of imaging, particularly computed tomography, in accurate diagnosis and differentiation from other common conditions.

## Introduction

As children begin to explore their surroundings, their mouths become active participants in this discovery process. Around 80% of reported cases of foreign body ingestion occur in pediatric patients, notably those aged between 6 months and 3 years [1]. Fortunately, many of these instances resolve spontaneously, with less than 1% demanding surgical intervention [1]. Moreover, the types of foreign objects ingested seem to correlate with cultural and geographical factors that influence dietary patterns, with Asian populations displaying a higher propensity for swallowing fish bones, for instance [1,2]. Generally, the ingestion of sharp objects carries a greater risk of complications such as ulceration, perforation, and the formation of fistulas or abscesses, potentially leading to increased morbidity and mortality [3]. In the United States, approximately 1500 deaths occur annually due to the ingestion of foreign objects [4]. Hence, prompt diagnosis and management are imperative to mitigate any potential adverse outcomes. Children pose a unique challenge in this regard due to their inability to effectively communicate their medical history, necessitating careful attention and thorough investigation. We illustrate this point with a case report about a 2-year-old boy who was initially misdiagnosed, resulting in a complex clinical course and the need for surgical intervention.

## Case presentation

A 2-year-old boy was brought to the emergency department due to intermittent abdominal pain over the past 8 days, primarily on the left side of his abdomen. Initially, the patient experienced mild pain, which was initially managed at a different institution under the diagnosis of constipation, with the administration of laxatives. However, over time, the intensity of the pain has progressively increased. There were no associated symptoms of vomiting or fever reported.

Upon admission, the patient appeared clinically unwell, yet afebrile and vitally except for tachycardia (110 beats/minute). Abdominal examination revealed a nondistended abdomen, with tenderness localized to the left side, encompassing both the left upper and left lower quadrants. A per rectal examination yielded unremarkable findings. Laboratory workup revealed leukocytosis with a predominance of neutrophils, alongside elevated inflammatory markers, while all other laboratory parameters were within normal ranges (Table 1).

**Table 1:** Summary of pertinent laboratory results before and after surgery.

Parameter	At admission	After surgery
White Blood Cells	16.20 × 10*3/uL	11.70 × 10*3/uL
Neutrophils	61.40 %	52.10 %
Erythrocyte sedimentation rate (ESR)	112 mm/h	21 mm/h
C-reactive protein (CRP)	66.91 mg/L	4 mg/L

**Fig. 1:**
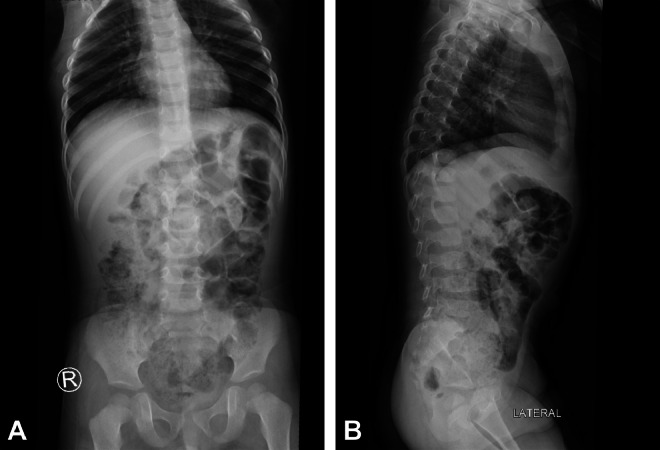
Supine anteroposterior (A) and lateral (B) X-ray images exhibit unremarkable findings.

**Fig. 2:**
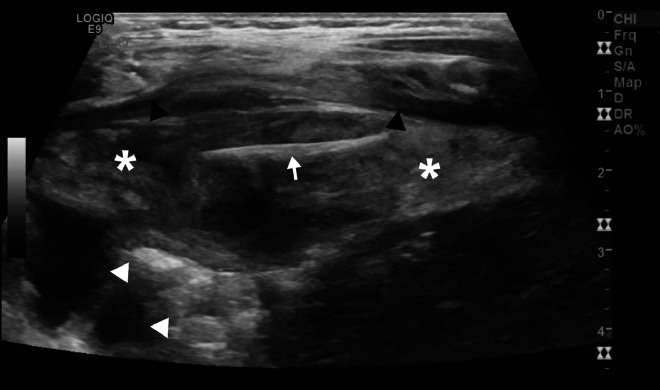
Abdominal ultrasonographic image at the left lower quadrant shows a well-defined fusiform-shaped, heterogeneous, predominantly hypoechoic mass (asterisks) resembling a localized fluid collection, measuring 7.0 × 2.5 cm (length x depth), is observed adjacent to the anterior abdominal wall (black arrowheads) and the colon (white arrowheads). Within this collection, there is a linear echogenic structure (arrow) measuring approximately 2.3 cm in maximum length, suggestive of a suspected foreign body.

**Fig. 3:**
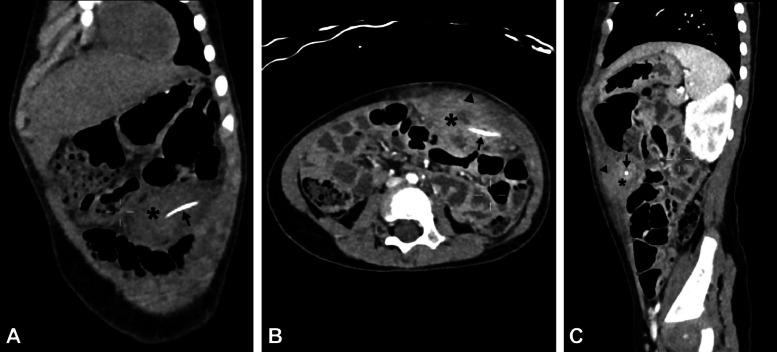
Coronal (A), axial (B), and sagittal (C) abdominal computed tomography images show at the left mid-abdominal region, a linear hyperdense foreign object (arrow), approximately 2.5 cm in length, is visibly lodged within the left intra-abdominal cavity (asterisk), characterized by heterogeneity. This cavity presents as a substantial localized collection, measuring 5.3 × 3.2 × 7.0 cm (craniocaudal × anteroposterior × transverse) in dimensions closely situated adjacent to the anterior abdominal wall (arrowheads).

Plain abdominal radiographs (Figs. 1A and B) showed no abnormalities, whereas ultrasound (US) imaging of the abdomen (Fig. 2) revealed a localized fluid collection on the left side containing a foreign body adjacent to the colon. Subsequently, abdominal computed tomography (CT) imaging (Figs. 3A-C) was conducted for further evaluation, revealing findings indicative of a complicated gastrointestinal perforation caused by ingestion of a foreign object, resulting in the formation of an abdominal abscess.

**Fig. 4:**
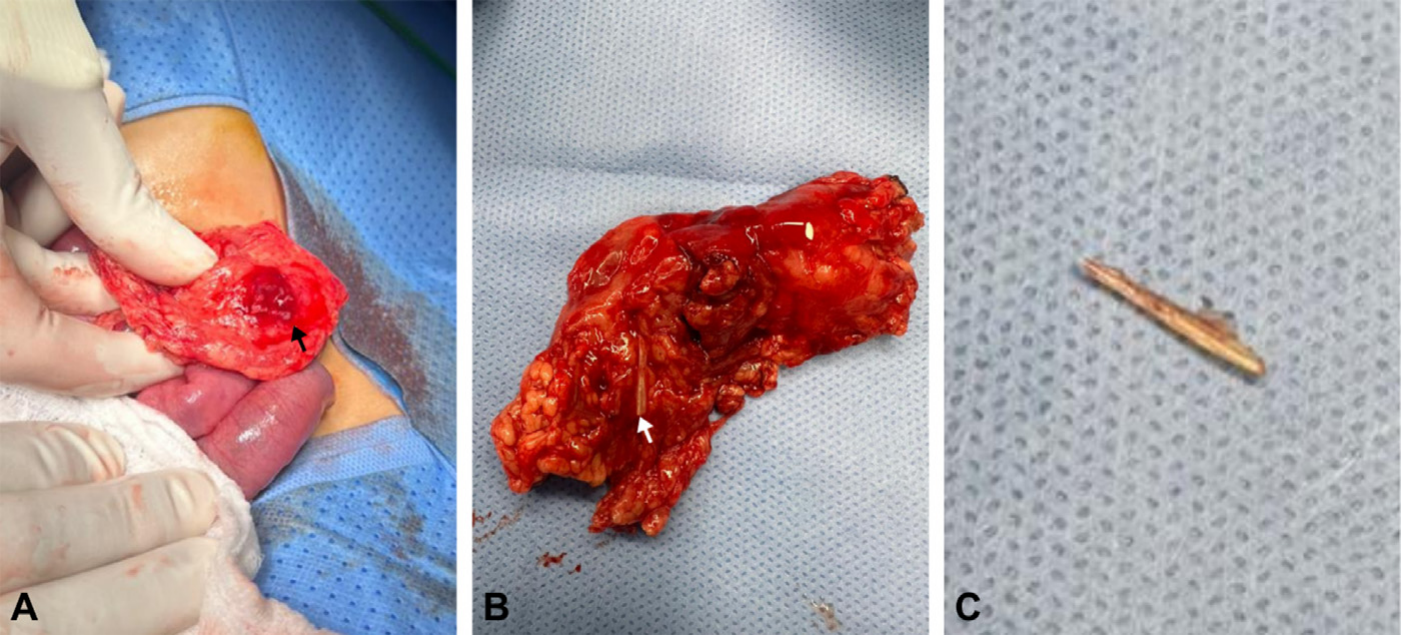
Gross intraoperative images. (A) part of the inflamed omentum (black arrow) adherent to the sealed perforation site forming a mass. (B) the resected omental mass with the dislodged foreign body (white arrow) visible. (C) the removed foreign body (i.e. fish bone).

The patient was admitted under pediatric surgery and an emergency laparotomy was performed. Surgical exploration showed a sealed left transverse colon perforation surrounded by omentum forming a mass (Fig. 4A) that was also adherent to the anterior abdominal wall. The remaining bowel segments were healthy. The omental abscess was surgically excised, revealing a contained collection of pus (∼ 5-7 mL), and a small piece of fish bone (Figs. 4B and C) surrounded by the omentum. The site of the identified sealed perforation was secured with a Lembert suture. Although the surrounding bowel segment exhibited slight inflammation, it remained viable, necessitating no resection of any bowel segments.

After surgery, the patient recovered smoothly with steady clinical improvement and favorable laboratory results (Table 1). Initially, he was kept NPO (nothing by mouth) and administered IV fluids, analgesics, and antibiotics (Ampicillin, Gentamicin, and Metronidazole) as part of standard postoperative care. On the fourth postoperative day, oral intake was initiated, and the patient tolerated it well. However, during the course of recovery, a superficial wound infection occurred, which was promptly addressed with local care and wound dressing. Despite this complication, the patient responded well to treatment and was discharged in good health on the eighth postoperative day. On outpatient follow-up, he was doing well with no active complaints.

## Discussion

While accidentally swallowing foreign bodies is not uncommon in children [1,5,6], the objects in question are usually coins, toys, and batteries [6]. In our case, a 2-year-old swallowed a fishbone, which is an odd occurrence in this age group. Perforations frequently occur in the terminal ileum in the small bowel and at the rectosigmoid junction in the large bowel due to changes in bowel caliber, the transition between mobile and fixed portions of the mesocolon, and acute angulations of the bowel at these locations [7,8]. However, in our case, the perforation occurred in the transverse colon. Individuals experiencing complicated foreign body ingestion may not recall the ingestion event and typically present with a spectrum of nonspecific clinical features contingent upon the site of perforation and lodgment of the offending object. These symptoms encompass abdominal pain, gastrointestinal manifestations like constipation, as well as constitutional symptoms such as fever [[9], [10], [11], [12]]. Hence, such cases could be misdiagnosed as appendicitis, perforated peptic ulcer, pancreatitis, or malignancy among other differentials [6,[13], [14], [15], [16]]. Nonetheless, patients may exhibit only delayed onset or subtle symptoms [11,15,17,18] or be asymptomatic [4,18], which can contribute to a delayed diagnosis and a more complex clinical course, given the challenge of establishing a clear connection between symptoms and the inciting event (i.e., foreign body ingestion), as observed in our case. Therefore, patients presenting with abdominal pain warrant thorough investigation for foreign body ingestion among other differentials, particularly in pediatric cases or those with pertinent medical history.

Given the varied clinical presentation of foreign body ingestion, coupled with the rarity of self-reporting and the relative infrequency of this condition compared to other differentials, imaging plays a crucial role in establishing diagnosis and guiding management [19,20]. Typically, plain radiographs poorly detect fish bones in the intestines as they are more likely to be radiolucent, or obscured by soft tissue [[20], [21], [22]] except in rare reported occasions [22,23]. Moreover, the presence of pneumoperitoneum is rare in gastrointestinal foreign body perforation [18] due to the gradual erosion by the foreign object, leading to covering of the perforation site, and limiting air passage. This limits the radiological significance of free air in both plain radiographs and CT imaging [7,20]. On the contrary, US and CT imaging are more reliable in diagnosing fish bone ingestion cases.

US excels at identifying intra-abdominal fluid and nonradiopaque foreign bodies like fishbones due to their high reflectivity, which produces background shadows. Moreover, alterations in surrounding tissues may signify underlying pathology [24]. US is also useful for diagnosing intra-abdominal abscesses containing fishbones, allowing for guided aspiration for diagnostic and treatment purposes [25]. In our case, US effectively depicted the fishbone and intra-abdominal fluid collection. Nonetheless, US efficacy is operator-dependent, and the quality of visualization may vary based on the depth of the perforation site [9].

CT imaging is the modality of choice as it offers heightened reliability (sensitivity 90%-100%; specificity 94%-100%) for managing fish bone ingestions, particularly in complicated scenarios like perforations [20] provided there is a high index of suspicion known to the radiologist [26]. Moreover, CT scans with thinner slices provide better foreign body detection and differentiation from blood vessels in contrast-enhanced studies [8,26]. CT provides improved characterization of the offending object and surrounding tissue and facilitates detection of complications which informs the selection of appropriate management interventions [8,20]. CT imaging also can be used to rule out other common misdiagnoses such as appendicitis and pancreatitis [6,13,27]. On CT scans, perforated bowel typically manifests with bowel wall thickening, mesenteric fat stranding, and localized pneumoperitoneum. However, these findings are nonspecific and should be correlated with the presence of a fishbone, which manifests as a linear calcified entity, to definitively establish the diagnosis [26]. On CT, the prevailing complication observed is the presence of abscesses, with intraperitoneal abscesses being the most frequently encountered type [22] manifesting as a localized fluid collection, as in our case. The utilization of contrast materials might cause difficulty in foreign body detection. Oral contrast may conceal the appearance of the fishbone in the intestinal lumen, while intravenous contrast enhances blood vessels that might resemble the fishbone's appearance [26]. However, contrast assists in delineating the bowel wall layers, extent of inflammation, and abscess formation [7,28]. While unenhanced CT images are more sensitive due to the natural contrast provided by fishbones against surrounding tissues, fishbones may still be identifiable in the postcontrast phase of contrast-enhanced studies if the CT imaging was primarily intended for other diagnostic considerations [28]. Nonetheless, surgical exploration might be necessary in cases of high clinical suspicion, as imaging can sometimes fail to detect foreign bodies [18,19,29,30].

Typically, incidents involving the ingestion of foreign bodies tend to resolve spontaneously without intervention within 1 week, especially once the object has moved beyond the esophagus [4,20,31]. However, the risk of complications remains notably heightened with sharp objects [4]. In cases of foreign body perforation, a proactive management strategy is implemented, customized based on the appropriateness of interventions, the patient's clinical status, imaging findings, and their response to treatment. This spectrum of approaches reported in the literature spans from conservative measures to more invasive surgical interventions.

In cases where imaging shows benign features (i.e. absence of complicated perforation), conservative management may be considered to manage them [19]. Documented cases effectively managed conservatively are available in the literature [8,19,32]. In contrast, conservative treatment was ineffective in other cases as their patient exhibited clinical improvement only following foreign body removal [14]. Generally, endoscopic removal is used in 10%-20% of cases [33] and varies in intestinal foreign body retrieval success rate, with some cases effectively treated [17,[34], [35], [36]] while others failed [37]. This approach is suitable when a portion of the perforating foreign body is situated within the lumen without complications requiring surgical intervention [[34], [35], [36]]. Surgical intervention is needed in less than 1% of foreign body ingestion cases [33] however is the mainstay of therapy for cases complicated by perforation. It employs various techniques, with suturing the site of perforation being the most frequently utilized method [12], as in our case. In our case, surgical intervention was necessary to manage the extraluminal migration of the fishbone and the associated abscess. The choice between laparotomy or laparoscopy hinges on clinical presentation, imaging findings, and surgical team expertise. While laparotomy is conventional, laparoscopy shows promise in select cases [9,29]. Postoperative recovery is typically smooth and uncomplicated for the majority of cases, with patients achieving full recovery within 1 week [6,13,27,30].

## Conclusion

Foreign body ingestion in pediatric patients poses challenges in both diagnosis and management due to its relatively rare occurrence, nonspecific presentation, and often delayed symptoms, leading to a disconnect between the incident and clinical manifestation. Therefore, foreign body ingestion should be considered among the differential diagnoses, particularly in high-risk populations such as children. As clinically elusive, imaging plays a pivotal role in diagnosing these cases and guiding management strategies to achieve optimal outcomes. Thus, a multidisciplinary approach and effective communication between clinicians and radiologists are essential. While various management strategies are documented in the literature, surgical intervention remains the cornerstone of treatment for fishbone ingestion complicated by bowel perforation.

## Data availability

Further data can be provided (if available) upon justified request. However, it is not publicly accessible due to privacy reasons.

## Patient consent

An informed consent was taken for the patient's parent for the publication of this case report.
